# High night-to-night variability in sleep apnea severity is associated with uncontrolled hypertension

**DOI:** 10.1038/s41746-023-00801-2

**Published:** 2023-03-30

**Authors:** Bastien Lechat, Kelly A. Loffler, Amy C. Reynolds, Ganesh Naik, Andrew Vakulin, Garry Jennings, Pierre Escourrou, R. Doug McEvoy, Robert J. Adams, Peter G. Catcheside, Danny J. Eckert

**Affiliations:** 1 Adelaide Institute for Sleep Health and FHMRI Sleep Health, College of Medicine and Public Health, Flinders University, Adelaide, Australia; 2 Baker Heart and Diabetes Research Institute, Melbourne, Australia; 3 Centre Interdisciplinaire du Sommeil, Paris, France

**Keywords:** Epidemiology, Diagnosis, Diagnostic markers

## Abstract

Obstructive sleep apnea (OSA) severity can vary markedly from night-to-night. However, the impact of night-to-night variability in OSA severity on key cardiovascular outcomes such as hypertension is unknown. Thus, the primary aim of this study is to determine the effects of night-to-night variability in OSA severity on hypertension likelihood. This study uses in-home monitoring of 15,526 adults with ~180 nights per participant with an under-mattress sleep sensor device, plus ~30 repeat blood pressure measures. OSA severity is defined from the mean estimated apnea–hypopnoea index (AHI) over the ~6-month recording period for each participant. Night-to-night variability in severity is determined from the standard deviation of the estimated AHI across recording nights. Uncontrolled hypertension is defined as mean systolic blood pressure ≥140 mmHg and/or mean diastolic blood pressure ≥90 mmHg. Regression analyses are performed adjusted for age, sex, and body mass index. A total of 12,287 participants (12% female) are included in the analyses. Participants in the highest night-to-night variability quartile within each OSA severity category, have a 50–70% increase in uncontrolled hypertension likelihood versus the lowest variability quartile, independent of OSA severity. This study demonstrates that high night-to-night variability in OSA severity is a predictor of uncontrolled hypertension, independent of OSA severity. These findings have important implications for the identification of which OSA patients are most at risk of cardiovascular harm.

## Introduction

Obstructive sleep apnea (OSA) is a common clinical sleep disorder characterized by repetitive upper airway collapse during sleep. OSA is estimated to affect approximately one billion people globally^1,2^. Untreated OSA is associated with a wide range of adverse health and safety consequences including increased risk of hypertension^3^, cardiovascular disease^4^, depression^5^, reduced quality of life^6^, traffic accidents^7^, and all-cause mortality^4^.

Recent evidence indicates that there is considerable night-to-night variation in OSA severity^2,8,9^. This has raised concerns regarding OSA misdiagnosis and possible misdirected management and care^2,8,9^. Night-to-night variation in OSA may explain, at least in part, why a single-night diagnosis of OSA shows inconsistent relationships with important health outcomes and responses to treatment^10–12^. Indeed, emerging evidence indicates that high night-to-night variability in OSA severity may be an important contributor to cardiovascular diseases such as atrial fibrillation^13,14^. Similarly, blood pressure varies widely from day-to-day in some people, and high day-to-day variability in blood pressure is associated with atrial fibrillation, OSA, all-cause mortality, and cardiovascular events, independent of mean blood pressure^15–21^. Whether night-to-night changes in OSA severity contribute to blood pressure variability is unknown.

To investigate the potential clinical importance of night-to-night variation in OSA severity on cardiovascular risk, this study was designed to determine the potential association between night-to-night variability in OSA severity and blood pressure using a validated under-mattress sleep monitor^2,22,23^ and a clinically validated home-blood pressure monitor. We hypothesized that high variability in OSA severity would be a strong predictor of hypertension and blood pressure variability.

This study includes data from 12,287 adults monitored over ~180 nights using an under-mattress sleep sensor device accompanied by ~30 repeat blood pressure measurements. Here we use the under-mattress sleep sensor technology to estimate OSA severity (mean apnea–hypopnea-index; AHI) and OSA variability (standard deviation of AHI over the recording period). People in the highest variability quartile within each OSA severity category have a 50–70% increased likelihood of uncontrolled hypertension compared to the lowest variability quartile, regardless of OSA severity. We conclude that high variability in OSA severity is an independent predictor of uncontrolled hypertension and this has major implications for identification of patients most at risk of cardiovascular harm. Furthermore, this study highlights the unique and important insights that multi-night, in-home, non-invasive monitoring of OSA severity can yield.

## Results

### Participant characteristics

Of the 15,526 users in the database, 1377 (8.9%) and 1482 (9.5 %) were excluded because they had <28 nights of sleep recordings or an average use of <4 times a week, respectively. A further 346 (2.2%) were excluded due to missing body mass index (BMI) data and 34 (0.2%) as they were <18 or >90 years old. The characteristics of the remaining 12,287 users are summarized in Table 1. The characteristics of the current participants were similar to a recent report that included data from >65,000 people to investigate multi-night OSA prevalence and single-night disease misclassification^2^. However, the proportion of males was slightly higher, and mean BMI and AHI values were ~1 kg/m^2^ and 3 events/h higher, respectively, in the current study sample (Supplementary Table 1). Most users resided in Europe (69%) and North America (27%). Participants had a mean (±SD) of 181 ± 69 overnight sleep recordings and median [IQR] 29 [12, 81] repeat blood pressure recordings. A total of 910,836 direct blood pressure entries were acquired during the recording period. 75% of the total blood pressure measurements were acquired as single time point measurements and 25% were acquired as the mean of three consecutive measurements.

**Table 1:** Baseline participant characteristics.

		Overall	No OSA	Mild OSA	Moderate OSA	Severe OSA
*n*		12,287	4529 (36.9%)	4233 (34.4%)	2256 (18.4%)	1269 (10.3%)
Age (years)		50 ± 12	44 ± 11	50 ± 11	55 ± 11	57 ± 11
BMI (kg/m^2^)		28 ± 6	27 ± 5	28 ± 5	30 ± 5	32 ± 6
Sex	Male	10,868 (88%)	3804 (84%)	3770 (89%)	2082 (92%)	1212 (96%)
	Female	1419 (12%)	725 (16%)	463 (11%)	174 (8%)	57 (4%)
Number of nights (*n*)		181 ± 69	179 ± 69	182 ± 69	183 ± 67	181 ± 70
Use per week, (nights/week)		6 ± 1	6 ± 1	6 ± 1	6 ± 1	6 ± 1
Apnea–hypopnea-index (events/h)	Mean	13 ± 14	2 ± 1	9 ± 3	21 ± 4	45 ± 14
	SD	6 ± 4	3 ± 1	6 ± 2	10 ± 3	15 ± 5
Number of BP entries m[IQR]		29 [12, 81]	24 [11, 67]	30 [12, 86]	34 [14, 95]	35 [14, 89]
Systolic BP (mmHg)	Mean	127 ± 12	124 ± 12	127 ± 12	129 ± 11	133 ± 12
	SD	9 ± 3	8 ± 3	9 ± 3	10 ± 3	10 ± 4
Diastolic BP (mmHg)	Mean	82 ± 8	80 ± 8	82 ± 8	83 ± 8	85 ± 9
	SD	6 ± 2	6 ± 2	6 ± 2	6 ± 2	7 ± 2
Hypertension	No	9820 (80%)	3922 (87%)	3376 (80%)	1700 (75%)	822 (65%)
	Yes	2467 (20%)	607 (13%)	857 (20%)	556 (25%)	447 (35%)

Data are reported as mean ± standard deviation (SD) for continuous variables and *n* (%) for categorical variables. Mean and SD for the apnea–hypopnea index values are calculated as the group average and standard deviation of the estimated apnea–hypopnea-index over the entire recording period available for each individual participant.

*BMI* body mass index, *BP* blood pressure.

**Fig. 1:**
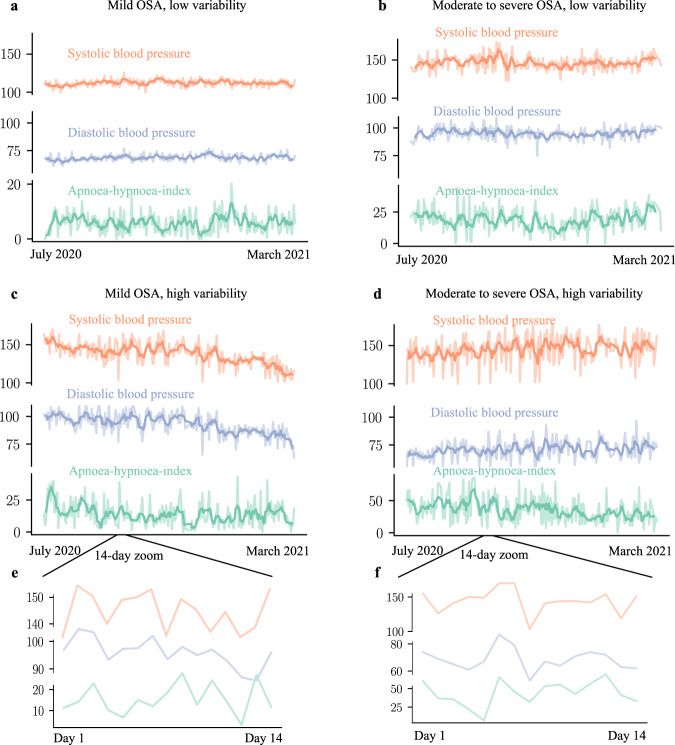
Nightly variation in systolic and diastolic blood pressure and estimated apnea–hypopnea index. Night-to-night variation in systolic (orange) and diastolic (blue) blood pressure and estimated apnea–hypopnea index (AHI; green) for participants with mild OSA (mean AHI > 5 but <15 events/h sleep) and either low (**a**) or high night-to-night variability (**c**) in AHI. Participants with moderate-to-severe OSA (mean AHI > 15 events) and either low (**b**) or high (**d**) night-to-night variability in AHI. **e** and **f** tracings represent a zoomed-in 14-day period of examples **c** and **d** (high night-to-night AHI variability) to show the temporal relationship between AHI and blood pressure variability. Darker lines are smoothed averaged over 7 days and light lines are raw data. Note: the temporal association between low night-to-night variability in AHI and blood pressure in panels **a** and **c**—and between high night-to-night variability in AHI and blood pressure in panels (**b**) and (**d**).

Mean systolic and diastolic blood pressure were 9 and 5 mmHg higher in people with severe versus no OSA (Table 1). Linear associations between OSA, OSA variability, and systolic and diastolic blood pressures (as a continuous variable), controlled for age, sex, and BMI also revealed that blood pressure was consistently higher (all *p*-values < 0.001) for participants with severe and variable OSA compared to controls and those with low night-to-night variability in OSA severity (see Tables 2 and 3, Supplementary Figs. 1 and 2). Individual examples of matched blood pressure and AHI measurements in people with and without high night-to-night variability in OSA severity and different OSA severity categories are displayed in Fig. 1 with additional examples shown in Supplementary Fig. 3.

**Table 2:** *β* coefficients (mean and 95% confidence interval) of the association between obstructive sleep apnea (OSA) severity with diastolic and systolic blood pressure.

OSA severity	No OSA	Mild OSA	Moderate OSA	Severe OSA
*Blood pressure*				
Systolic	0 (ref)	1.15 (0.68, 1.61)	1.92 (1.34, 2.51)	3.32 (2.58, 4.05)
Diastolic	0 (ref)	1.81 (1.48, 2.15)	2.43 (2.00, 2.85)	3.24 (2.70, 3.78)

OSA severity categories were defined using standard clinical cut-offs of the apnea–hypopnea index (<5 = no OSA, ≥5 and <15 = mild, ≥15 and <30 = moderate and ≥30 events/h sleep = severe OSA). Models were adjusted for age, sex, and BMI.

**Table 3:** *β* coefficients (mean and 95% confidence interval) of the association between quartiles of night-to-night variability in obstructive sleep apnea (OSA) severity with diastolic and systolic blood pressure.

OSA variability	Quartile 1	Quartile 2	Quartile 3	Quartile 4
*Blood pressure*				
Systolic	0 (ref)	0.32 (−0.23, 0.86)	1.42 (0.82, 2.02)	2.06 (1.22, 2.89)
Diastolic	0 (ref)	1.16 (0.76, 1.56)	2.26 (1.82, 2.69)	2.41 (1.80, 3.02)

OSA variability was defined as average standard deviation of the apnea–hypopnea index and categorized using quartiles. Models were adjusted for age, sex, and BMI and mean apnea–hypopnea-index.

### OSA and uncontrolled hypertension

**Fig. 2:**
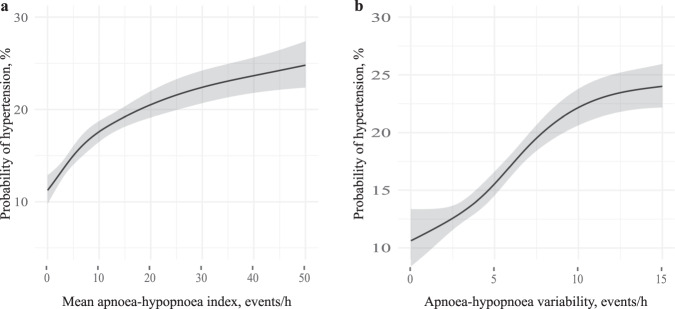
Association between mean apnea–hypopnea index, apnea–hypopnea index variability and hypertension. Marginal probability of hypertension in relation to: **a** mean apnea–hypopnea index and **b** apnea–hypopnea index variability.

OSA severity (estimated AHI mean 75th vs. 25th centile: 16.8 vs. 3.0 events/h; OR [95% CI], 1.54 [1.38, 1.72]) and higher variability in OSA severity (estimated AHI SD 75th vs. 25th centile: 8.2 vs. 3.4; 1.63 [1.46, 1.83]) were associated with increased uncontrolled hypertension likelihood in separate models after controlling for age, BMI, and sex. Both associations were nonlinear, and marginal probability plots are shown in Fig. 2a, b. There were no significant interactions between sex and OSA severity or sex and OSA variability (*p*-values = 0.75 and 0.73, respectively). When OSA severity and variability were combined into one model, the variance inflation factors (a measure of collinearity) for OSA severity and variability were below 5 (2.81 and 2.74), supporting the combination of both metrics into a single model. The likelihood ratio test indicated that a model with both OSA severity and variability provided a superior model fit than a model with OSA severity alone (*p*-value < 0.001). In this combined model, OSA severity was associated with a 7% increase in uncontrolled hypertension likelihood (1.07 [1.00, 1.14]). Furthermore, OSA variability independent of OSA severity was associated with a 51% increase (1.51 [1.31, 1.75]) in uncontrolled hypertension likelihood.

**Fig. 3:**
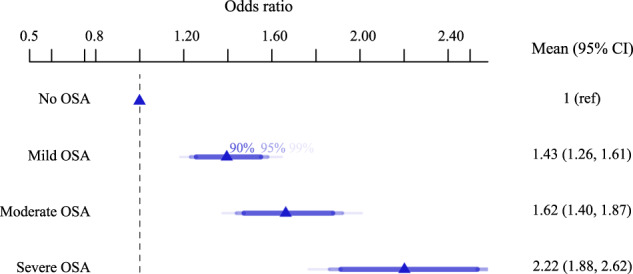
Association between obstructive sleep apnea (OSA) severity with uncontrolled hypertension. Odds ratio (Estimate and 95% confidence interval) of the association between obstructive sleep apnea (OSA) severity with hypertension. This model is controlled for age, sex, and BMI. OSA category was defined using standard clinical cut-offs of the apnea–hypopnoea index where <5 = no OSA, ≥5 and <15 = mild, ≥15 and <30 = moderate and ≥30 events/h sleep = severe OSA).

**Fig. 4:**
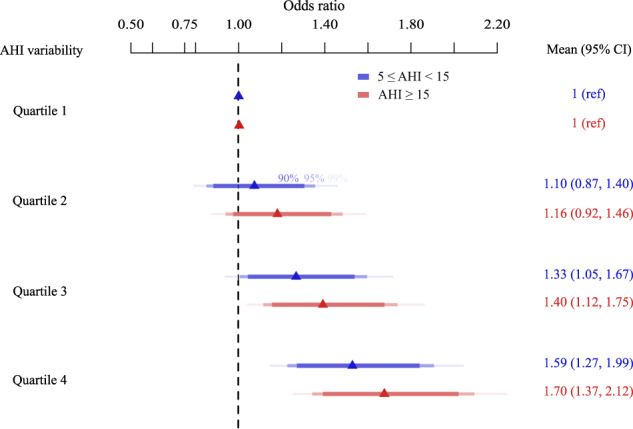
Association between nightly variation in obstructive sleep apnea severity with uncontrolled hypertension. Odds ratio (mean and 95% confidence interval) of the association between obstructive sleep apnea severity as measured by the mean apnea–hypopnea index (AHI), and night–night variability in AHI, measured by the SD of AHI, with uncontrolled hypertension. Mild OSA is defined as an AHI between 5 and 15 events/h. Moderate/severe OSA is defined as an AHI > 15 events/h. Within each OSA severity category, the lowest quartiles of variability in AHI are taken as the reference.

Categorical analysis of OSA severity confirmed that mild, moderate, and severe OSA were associated with increased uncontrolled hypertension likelihood compared to the no OSA group (Fig. 3). Participants with mild, moderate, and severe OSA were at 43% (OR 95%CI; 1.43 [1.26–1.61]), 62% (1.62 [1.40–1.87]) and 122% (2.22 [1.88–2.62]) increased uncontrolled hypertension likelihood versus no OSA. Direct comparisons between the association of OSA severity, as measured over multiple nights with the Withings Sleep Analyzer (WSA), with hypertension in the current study versus single-night assessment in the Sleep Heart Health Study are presented in Supplementary Table 2. Furthermore, analysis of OSA variability by quartiles within each traditionally defined OSA severity category, indicated that higher night-to-night variability in OSA severity was associated with greater uncontrolled hypertension likelihood (Fig. 4).

After excluding blood pressure entries not taken during the morning, 5383 (43.7%) participants remained for the sensitivity analysis. Of these, 1170 (21.7%) cases of hypertension were observed. In separate models, high average AHI (OR [95% CI]; 1.51 [1.29, 1.77]) and high AHI variability (1.58 [1.33, 1.88]) were associated with an increased risk of hypertension. In the combined model, average AHI was associated with a 14% increase in hypertension risk (1.14 [1.02, 1.27]), and variability in AHI was associated with a 36% increase in hypertension risk (1.36 [1.09, 1.69]). Model fit was superior in the combined model than in the AHI-mean-only model (*p* = 0.029). Quartile analysis of AHI variability within the OSA severity category also suggested increased hypertension risk for participants with variable AHI, although some results became non-significant likely due to the smaller sample size (Supplementary Table 3). Findings were also similar when the model was additionally adjusted for the number of blood pressure entries (Supplementary Tables 4 and 5) and average total sleep time (Supplementary Tables 6 and 7). Three alternative cut-offs to define uncontrolled hypertension also did not change the main findings (Supplementary Tables 8 and 9).

### Mediation analysis

Mediation analysis was consistent with a mediating effect of OSA night-to-night variability in the association between OSA severity and uncontrolled hypertension (Supplementary Table 10). A 1-unit increase in AHI mean was associated with a 0.9% increase in the probability of having uncontrolled hypertension (similar effect size as observed in Fig. 2a). The increase was due to both a direct effect of mean AHI on uncontrolled hypertension (0.4% increase), but also via an increase in night-to-night AHI variability (indirect effect, 0.5%). About 55% (0.5/0.9) of the effect of mean AHI on uncontrolled hypertension is mediated by an increase in AHI variability. There are important limitations associated with this mediation analysis, as highlighted in the Supplementary Discussion.

### OSA and blood pressure variability

Similar to the OSA and uncontrolled hypertension findings, quartile analyses indicated an association between OSA variability, OSA severity categories, and greater blood pressure variability over the ~6 months recording period. In the combined model, an increase in AHI variability was associated with a ~0.27 mmHg increase in diastolic blood pressure variability (75th vs. 25th; *β* [95% CI], 0.27 [0.19, 0.35]) and a ~0.37 mmHg increases in systolic blood pressure variability (0.37 [0.26, 0.48]) independent of mean AHI. In the combined model, mean AHI was not associated with blood pressure variability. In separate models, mean AHI (systolic: 0.31 [0.24, 0.37]; diastolic: 0.24 [0.19, 0.28]) and AHI variability (systolic: 0.41 [0.34, 0.48]; diastolic: 0.31 [0.26, 0.36]) were associated with increased blood pressure variability. The combined model provided a superior model fit compared to a model with mean AHI alone (*p*-value < 0.001). Quartile analyses also indicated an association between OSA severity categories, OSA variability, and greater blood pressure variability. Severe OSA was associated with ~1 mmHg increase in systolic and ~0.7 mmHg increase in diastolic blood pressure variability compared to no OSA (Supplementary Table 11). Furthermore, compared to the lowest quartile, the highest quartile of OSA variability was associated with an increase in both systolic (~0.5 to 1 mmHg) and diastolic (~0.4 to 0.8 mmHg) blood pressure variability independent of OSA severity category (Supplementary Table 12). Limiting the analyses to only morning blood pressure recordings did not change any of the main findings (Supplementary Table 13).

## Discussion

The current findings demonstrate an association between high night-to-night variability in OSA severity with increased uncontrolled hypertension risk and blood pressure variability. Even after accounting for the OSA severity category, OSA variability itself was a strong independent predictor of both uncontrolled hypertension and blood pressure variability. These new findings provide important insight into the different manifestations of OSA and its consequences and highlight the need to consider the importance of night-to-night variability in disease severity. The current multi-night recordings to quantify OSA with a simple non-invasive under-mattress sensor in the home, also support previously documented associations between OSA severity and hypertension risk from conventional single-night polysomnography recordings^3,24,25^. Accordingly, there is considerable potential to incorporate new simplified monitoring approaches to aid current single-night diagnostics.

The current findings are in accordance with emerging evidence that indicates an association between cardiovascular disease and night-to-night variability in OSA severity^13,14^. A key mechanism through which OSA and cardiovascular risk may be related is via increased day-to-day blood pressure variability^20,21,26^. Blood pressure variability has been associated with an increased risk of cardiovascular events, all-cause mortality, vascular organ damage, atrial fibrillation, and dementia^15–19,27^. Consistent with the current findings of increased blood pressure in those with severe or variable OSA, day-to-day blood pressure variation of similar magnitude to the current study is associated with increased all-cause mortality and non-fatal and fatal cardiovascular events^28^. The increased risk of hypertension and blood pressure variability may reflect a direct physiological increased risk from people with a “variable OSA phenotype”. The multifaceted nature of OSA pathophysiology may place some people more prone to night-to-night variation in OSA severity. These concepts, and the potential underlying mechanisms, warrant further investigation.

Our findings also highlight the important new information that multi-night monitoring of OSA and blood pressure can yield. Current one-size-fits-all clinical care approaches based on a single-night diagnostic study may not be appropriate for people with high internight variability and may explain, at least in part, the heterogeneity in prior treatment trials^12,29,30^. More in-depth assessment of hypoxemia, OSA endotypes, insomnia, and sleep fragmentation may be valuable in more complex manifestations of OSA^31–33^. The findings of this study also warrant future prospective trials to investigate the effects of different sleep patterns and night-to-night variability in OSA severity and the effects of different therapies on other key cardiovascular and health consequences such as mental health, sleepiness, workplace and traffic accident, and cognitive impairment and their potential interactions.

The volume of physiological data available from this study is substantially greater than previous cohort studies that have investigated OSA and its consequences^3,24,25^. The large sample size allows for greater precision around estimates of OSA severity and uncontrolled hypertension risk for increased power to detect relationships with available exposure variables. The non-invasive monitoring technology used in this study allowed us to identify relationships between variability in OSA severity and blood pressure in a large number of participants over a prolonged period, which is simply not feasible with conventional sleep monitoring approaches. Data were also collected in the participant’s home environment, more directly relevant to real-world risk exposure conditions compared to clinical laboratory sleep study settings.

However, AHI derived from the under-mattress sensor includes fewer input variables in which to detect respiratory events compared to conventional polysomnography. Accordingly, the possible contribution of other physiological aspects of OSA (e.g., hypoxia) that may contribute to hypertension risk and blood pressure variability requires further consideration. However, validation studies versus gold standard polysomnography in over 150 participants^2,22^ support the device performance characteristics. Nonetheless, the potential effects of comorbid insomnia, medications, and other clinical covariates on device accuracy remain to be investigated. Despite these potential unknown influences, OSA prevalence estimates using, non-contact multi-night data yield very similar findings to previously published literature^1,2^. Similarly, misclassification rates and AHI variability are comparable to data derived from multi-night in-laboratory polysomnography and other home sleep apnea tests^2,8,9^. The effect size of the association between the estimated AHI and uncontrolled hypertension detected in the current study is also similar to existing epidemiological trials such as the Sleep Heart Health Study, the Wisconsin cohort, and the HypnoLaus cohort^25,34,35^. While the magnitude of the effect size was comparable between studies, it is important to note that, unlike the current investigation, prior epidemiological studies also included the use of antihypertensive medications in the definition of hypertension. Thus, these findings provide support that the multi-night mean AHI estimates derived in the current study provide comparable, and potentially superior insight, into key health outcomes such as hypertension versus traditional single-night but more complex polysomnography approaches.

Instructions on the timing of blood pressure measurements were not given to participants. Thus, some of the variability in blood pressure may reflect circadian changes across the day^28^. However, the main study findings remained in sensitivity analyses when limited to morning blood pressure entries. Accordingly, circadian influences appear unlikely to have been a major confounder in the current study. Indeed, consistent with previous findings, variability in systolic blood pressure is a significant predictor of death when blood pressure is measured in the morning or evening, or both^16^. The number of clinical covariates available in this study was also somewhat limited. Thus, the potential impact of uncontrolled confounding behavioral and lifestyle factors (diet, exercise, alcohol, caffeine, tobacco use, and medications)^36,37^ and treatment status to influence the current findings remain to be investigated. Like many digital health innovations and consumer-data research, there is a balance between data volume and the ability to capture all clinically relevant variables. Conceptually, these data could also be synchronized via electronic health records databases, and via the development of new digital health tools to automatically capture more diverse aspects of a person’s health. Multimodal inputs to predictive algorithms (i.e., using health data from different sources) have been recently shown to better predict health outcomes compared to single-source approaches across 12 predictive tasks, including 10 distinct chest pathology diagnoses, hospital length-of-stay, and 48 h mortality predictions^38^. Similarly, the field of sleep medicine may benefit from multi-input clinical data where daytime symptoms, overall clinical history, and multi-sensor recordings could be used to better predict health outcomes and treatment response.

The decision of the users to purchase a WSA and monitor their blood pressure may represent a selection bias towards those who had preexisting sleep and/or cardiovascular problems. Indeed, users were predominantly male, clearly indicating a sex-specific participation bias. Nonetheless, while no sex differences were detected in the current study in relation to the detected associations with uncontrolled hypertension, the pathophysiology of OSA differs in women versus men^39^. Thus, it will be important to investigate whether the potential consequences of night-to-night variability in OSA severity are comparable between sexes in a larger cohort with more comparable numbers of women.

Despite these methodological considerations, the identification of strong associations between blood pressure variability, uncontrolled hypertension, and high night-to-night variability in OSA severity provides further support for the unique and important insights that multi-night non-invasive monitoring can yield regarding clinical end-points and potential health risks.

## Methods

### Participants

Data were acquired retrospectively from 15,526 participants from a consumer-user database of people aged between 18 and 90 years who purchased an under-mattress sleep sensor device WSA and reported blood pressure measurements on at least five separate occasions. Blood pressure measurements were acquired using an FDA-registered Withings blood pressure cuff monitor in the participant’s home, synchronized directly to the Withings database (both devices are CE-medical IIa certified). Data were collected between 1 July 2020 and 30 March 2021. Further inclusion criteria included ≥28 nights of WSA recordings and an average use of ≥4 times per week. All participants provided written consent through the Withings app for their deidentified data to be used for research purposes when signing up for a Withings account and the current study was approved by the Flinders University Human Research Ethics Committee.

### Monitoring equipment

The WSA is a nearable sleep monitoring device placed under-the-mattress that detects body movements, respiratory rate, heart rate, snoring, and cessation of breathing episodes. These signals are used to estimate the AHI using automated algorithms that show good agreement with in-laboratory polysomnography-derived AHI with high predictive performance to classify mild (89% sensitivity and 75% specificity), moderate-to-severe (88% sensitivity and 88% specificity) and severe OSA (86% sensitivity and 91% specificity)^2,22,23^. The estimated AHI also has minimal bias with in-laboratory polysomnography-derived AHI when the AHI is considered as a continuous variable^22^.

The Withings blood pressure monitor used in conjunction with the WSA comes with an instruction booklet that outlines how to take a blood pressure measurement. The user is instructed to (1) rest for ≥5 min before taking a measurement, (2) be seated in a comfortable position and in a quiet area with legs uncrossed, feet flat on the floor, and back/arm supported (3) not speak during the measurements, and (4) perform the measurement on their left arm. The user has the option of performing a single measurement or taking three consecutive measurements to acquire an average blood pressure value.

### Effect of OSA on uncontrolled hypertension and potential confounders

Hypertension exposure variables were the mean estimated AHI (hereafter referred to as “OSA severity”) and the average standard deviation of estimated AHI (hereafter termed “OSA variability”). OSA severity and variability were calculated for each participant derived from all available nights for each individual. OSA severity categories were defined using standard clinical cut-offs^40^ (<5 = no OSA, ≥5 and <15 = mild, ≥15 and <30 = moderate and ≥30 events/h sleep = severe OSA), and OSA variability was defined within each OSA severity category.

Uncontrolled hypertension was defined as a mean systolic blood pressure ≥140 mmHg or mean diastolic blood pressure ≥90 mmHg^41^ across the monitoring period as these thresholds are most commonly applied in previously published papers on the relationship between uncontrolled hypertension and OSA severity^3,25,34,35^. However, alternate definitions of uncontrolled hypertension were also included in sensitivity analyses. Only participants with five or more independent blood pressure measurements across the assessment period were included in the analysis. Systolic and diastolic blood pressure variability was defined as the standard deviation of the systolic and diastolic blood pressure across all measurements during the recording period.

All first-time users of the WSA are prompted to enter their age, sex, height, and weight from which body mass index (BMI) is calculated. These data were either self-reported and manually entered or, in the case of weight, acquired via Withings scales, a device fed directly into the online Withings database.

### Statistical analysis

Odds ratio (ORs) and 95% confidence interval (CIs) were determined using logistic-regression models to assess the association between OSA severity, OSA variability, and uncontrolled hypertension. Linear regression was used to assess the association between OSA severity, OSA variability, and blood pressure variability. Arbitrary cut-offs for continuous variables were omitted in favor of restricted cubic spline transformations, which are better suited to non-linearity. Thus, ORs (or *β* coefficients) for continuous variables were used to compare the 75th percentile to that of the 25th percentile, using the 25th percentile as the reference.

OSA severity and variability were first included in separate models (Model 1A and 1B), and then in a combined model (Model 2). All models also included age, BMI, and sex. Likelihood ratios were used to compare predictive performance between models. Collinearity was assessed using variance inflation factors. Interactions between OSA severity and OSA variability and sex were also examined. Although the primary analysis examined OSA severity as a continuous measure, secondary analyses also examined OSA severity categorized according to standard clinical cut-offs and quartiles. Logistic and linear regression was performed in the R programming language, using the rms modeling package^42^.

### Sensitivity analyses

Four sensitivity analyses were conducted to further validate our findings. Firstly, given that blood pressure may vary during the day, we performed a sensitivity analysis where only morning blood pressure, measured between 6 a.m. and 12 p.m., entries were included. Secondly, the analysis was repeated by further adjusting for the number of blood pressure entries to control for potential biases associated with a variable number of blood pressure entries for each participant. Third, total sleep time has been shown to influence AHI certainty^43^, and was therefore included as a confounder in the third sensitivity analysis. Finally, alternative definitions of hypertension were also investigated, including the home-blood pressure cut-offs of the European/NICE guidelines^44,45^ (systolic blood pressure/diastolic blood pressure ≥ 135/85), the AHA guidelines (33, 34) (≥130/80) and stage 2 hypertension of the NICE guidelines^44^ (≥150/95).

### Mediation analysis

A potential mediating effect of night-to-night variability in OSA severity in the association between OSA severity and uncontrolled hypertension was investigated using a model depicted in Supplementary Fig. 4. The model was estimated using a diagonally weighted least-squares approach with a probit link function and all associations were adjusted for age, BMI, and sex. The analysis was conducted using the Lavaan package^46^ in R programming language.

### Reporting summary

Further information on research design is available in the Nature Research Reporting Summary linked to this article.

## Supplementary information

Supplementary material

REPORTING SUMMARY

## Data Availability

Deidentified data that support the findings of this study, including individual data, are available from the corresponding author upon request subject to ethical and data custodian (Withings) approval.

## Code availability

Code for the analysis is available upon reasonable request from the corresponding author.
