# Supplementary material for: High night-to-night variability in sleep apnea severity is associated with uncontrolled hypertension
Source: NPJ Digit Med. 2023 Mar 30;6:57. doi: 10.1038/s41746-023-00801-2 (PMC10060245; doi:10.1038/s41746-023-00801-2)
Supplement: Supplementary file 1 — Supplementary material [file 41746_2023_801_MOESM1_ESM.pdf]

Supplementary material: High night-to-night variability in sleep apnoea severity is associated with uncontrolled hypertension.

Supplementary Tables

Supplementary Table 1: *Comparison between participants' characteristics included in this study and participants' characteristics from an earlier report [1].*

		Overall	Excluded	Included
**N**		67,278	54,991	12,287
**Age** (years)		47 ±13	46 ±13	50 ±12
**BMI** (kg/m^2)		27 ±5	27 ±5	28 ±6
**Sex**	**Male**	52,533 (78%)	41,665 (76%)	10,868 (88%)
	**Female**	14,745 (22%)	13,326 (24%)	1,419 (12%)
**Number of nights** (n)		174 ±72	172 ±69	181 ±69
**Apnea-hypopnea-index** (events/h)	**Mean***	11 ±13	10 ±13	13 ±14
	**SD***	6 ± 4	6 ±4	6 ±4
**Mean total sleep time** (min)		448 ± 50	449 ± 50	446 ± 49

Supplementary Table 2: *Adjusted Odds Ratio (OR) and 95% Confidence Intervals (CIs) of the association between hypertension and the apnoea-hypopnoea index in the Sleep Heart Health Study (SHHS) and in our study. In the Sleep Heart Health Study, hypertension was defined as systolic blood pressure ≥ 140 or diastolic blood pressure ≥ 90 or use of hypertensive medications [2]. The OSA severity categories were defined as follow: (<1.5, ≥1.5 and <5; ≥5 and <15; ≥15 and <30; ≥30 events/h). Models are adjusted for age, sex and BMI. The SHHS model is additionally adjusted for ethnicity.*

	OR (95%CI)	
AHI category	SHHS[2]	This study
<1.5	1.00	1.00
≥1.5 and <5	1.12 (0.96-1.30)	1.20 (1.00-1.44)
≥5 and <15	1.28 (1.09-1.48)	1.56 (1.31-1.86)
≥15 and <30	1.32 (1.08-1.61)	1.82 (1.51, 2.20)
≥30	1.60 (1.23-2.08)	2.48 (2.02, 3.06)

Supplementary Table 3*: Odds ratio (Mean and 95% confidence interval) of the association between obstructive sleep apnoea severity and variability with hypertension. Mild OSA is defined as an apnoea hypopnoea index (AHI) between 5 and 15 events/h. Moderate/Severe OSA is defined as an AHI of more than 15 events/h. Within each OSA severity category, the lowest quartiles of variability in AHI are taken as the reference.*

	OSA variability			
**OSA severity category**	**Quartile 1**	**Quartile 2**	**Quartile 3**	**Quartile 4**
Mild OSA	1 (ref)	1.34<br>(0.96, 1.88)	1.22<br>(0.87, 1.71)	1.44<br>(1.03, 2.01)
Moderate to severe OSA	1 (ref)	1.12<br>(0.82, 1.53)	1.39<br>(1.02, 1.90)	1.19<br>(0.87, 1.63)

Supplementary Table 4: *Association between OSA and uncontrolled hypertension with (model 2) and without additional adjustment for total sleep time (model 1). Models are also adjusted for age and body mass index.*

	Model 1	Model 2
No OSA	1 (ref)	1 (ref)
Mild OSA	1.43 (1.26, 1.61)	1.42 (1.26, 1.61)
Moderate OSA	1.62 (1.40, 1.87)	1.62 (1.40, 1.87)
Severe OSA	2.22 (1.88, 2.62)	2.22 (1.88, 2.62)

Supplementary Table 5*: Association between night-to-night variability in OSA severity and uncontrolled hypertension, with (model 2) and without additional adjustment for the number of blood pressure entries (model 1). Models are also adjusted for age and body mass index.*

	Model 1	Model 2
**Mild OSA**		
Quartile 1	1 (ref)	1 (ref)
Quartile 2	1.1 (0.87, 1.40)	1.09 (0.87, 1.39)
Quartile 3	1.33 (1.05, 1.67)	1.32 (1.05, 1.67)
Quartile 4	1.59 (1.27, 1.99)	1.59 (1.27, 1.99)
**Moderate to severe OSA**		
Quartile 1	1 (ref)	1 (ref)
Quartile 2	1.16 (0.92, 1.46)	1.16 (0.92, 1.46)
Quartile 3	1.4 (1.12, 1.75)	1.4 (1.12, 1.75)
Quartile 4	1.7 (1.37, 2.12)	1.7 (1.37, 2.12)

Supplementary Table 6*: Association between OSA severity and uncontrolled hypertension with (model 2) and without additional adjustment for total sleep time (model 1). Models are also adjusted for age and body mass index.*

	Model 1	Model 2
No OSA	1 (ref)	1 (ref)
Mild OSA	1.43 (1.26, 1.61)	1.43 (1.27, 1.61)
Moderate OSA	1.62 (1.40, 1.87)	1.62 (1.40, 1.87)
Severe OSA	2.22 (1.88, 2.62)	2.20 (1.87, 2.60)

***Supplementary Table** 7: Association between night-to-night variability in OSA severity and uncontrolled hypertension, with (model 2) and without additional adjustment for total sleep time (model 1). Models are also adjusted for age and body mass index.*

	Model 1	Model 2
**Mild OSA**		
Quartile 1	1 (ref)	1 (ref)
Quartile 2	1.1 (0.87, 1.40)	1.11 (0.87, 1.40)
Quartile 3	1.33 (1.05, 1.67)	1.33 (1.06, 1.67)
Quartile 4	1.59 (1.27, 1.99)	1.6 (1.27, 2.00)
**Moderate to severe OSA**		
Quartile 1	1 (ref)	1 (ref)
Quartile 2	1.16 (0.92, 1.46)	1.16 (0.92, 1.45)
Quartile 3	1.4 (1.12, 1.75)	1.38 (1.10, 1.73)
Quartile 4	1.7 (1.37, 2.12)	1.68 (1.34, 2.10)

***Supplementary Table* 8**: *Odds ratio (Mean and 95% confidence interval) of the association between obstructive sleep apnoea (OSA) severity (apnoea/hypopnea index- AHI mean) and variability (AHI variability) with multiple definitions of hypertension. Continuous variables were modelled using restricted cubic spline transformations. Thus, odds ratios (or β coefficients) for continuous variables were used to compare the 75th percentiles to that of the 25th percentile, using the 25th percentile as the reference. OSA severity and variability were first included in separate models (Model 1A and 1B), and then in a combined model (Model 2).*

		**Model 1A**	**Model 1B**	**Model 2**	
**Hypertension definition**	**Hypertension prevalence, %**	AHI mean	AHI variability	AHI mean	AHI variability
SBP ≥ 135 or DBP ≥85	39.5%	1.50 (1.37, 1.64)	1.59 (1.45, 1.76)	1.06 (0.99, 1.14)	1.49 (1.31, 1.69)
SBP≥130 or DBP≥80	63%	1.41 (1.29, 1.56)	1.48 (1.33, 1.64)	1.06 (0.97, 1.15)	1.39 (1.21, 1.59)
SBP≥150 or DBP≥95	7.2%	1.63 (1.38, 1.93)	2.00 (1.68, 2.38)	1.16 (1.07, 1.26)	1.65 (1.34, 2.03)

Supplementary Table 9: *Odds ratio (Mean and 95% confidence interval) of the association between obstructive sleep apnoea severity and variability with multiple definition of hypertension. Mild OSA is defined as an apnoea/hypopnoea index (AHI) between 5 and 15 events/h. Moderate/Severe OSA is defined as an AHI of more than 15 events/h. Within each OSA severity category, the lowest quartiles of variability in AHI are taken as the reference.*

		OSA variability		
**OSA severity category**	**Quartile 1**	**Quartile 2**	**Quartile 3**	**Quartile 4**
**Hypertension defined as SBP ≥ 135 or DBP ≥85 mmHg [3, 4]**				
Mild OSA	1 (ref)	1.08 (0.90, 1.29)	1.34 (1.11, 1.60)	1.42 (1.18, 1.71)
Moderate to severe OSA	1 (ref)	1.25 (1.03, 1.51)	1.47 (1.21, 1.78)	1.56 (1.29, 1.92)
**Hypertension defined as SBP≥130 or DBP≥80 mmHg [5, 6]**				
Mild OSA	1 (ref)	0.97 (0.81, 1.16)	1.37 (1.14, 1.65)	1.41 (1.17, 1.71)
Moderate to severe OSA	1 (ref)	0.99 (0.81, 1.22)	1.33 (1.07, 1.65)	1.36 (1.09, 1.70)
**Hypertension defined as SBP≥150 or DBP≥95 mmHg [3]**				
Mild OSA	1 (ref)	1.42 (0.96, 2.11)	1.67 (1.13, 2.45)	2.02 (1.40, 2.94)
Moderate to severe OSA	1 (ref)	1.26 (0.90, 1.76)	1.40 (1.01, 1.94)	1.70 (1.24, 2.33)

Supplementary Table 10*: Mediation analysis. The direct effect is the effect of OSA severity alone on uncontrolled hypertension. The indirect effect is the effect of OSA severity on uncontrolled hypertension via a potential increase in night-to-night variability of OSA severity. The total effect is the overall effect of OSA severity on uncontrolled hypertension.*

	Estimate	95% CI	z-value	p-value
Direct effect	0.4	[0.1, 0.7]	2.6	0.01
Indirect effect	0.5	[0.3, 0.8]	4.7	< 0.001
Total effect	0.9	[0.7, 1.1]	9.5	< 0.001

Supplementary Table 11: *Estimated marginal means (parameter estimate and 95% confidence interval) of the association between obstructive sleep apnoea (OSA) severity with diastolic and systolic blood pressure variability. OSA severity categories were defined using standard clinical cut-offs of the mean apnoea-hypopnoea index (< 5 = no OSA, ≥ 5 and < 15 = mild, ≥ 15 and < 30 = moderate, and ≥ 30 events/h sleep= severe OSA).*

	OSA severity category			
**Blood pressure variability**	No	Mild OSA	Moderate OSA	Severe OSA
**Systolic**	0 (ref)	0.23<br>(0.09, 0.37)	0.47<br>(0.29, 0.65)	0.95<br>(0.72, 1.17)
**Diastolic**	0 (ref)	0.20<br>(0.10, 0.30)	0.43<br>(0.30, 0.55)	0.72<br>(0.56,0.87)

Supplementary Table 12: *Estimated marginal means (parameter estimate and 95% confidence interval) of the association between obstructive sleep apnoea (OSA) severity and variability with diastolic and systolic blood pressure variability. Mild OSA is defined as a mean apnoea hypopnoea index (AHI) between 5 and 15 events/h. Moderate to severe OSA is defined as a mean AHI of > 15 events/h. Within each OSA severity category, the lowest quartiles of variability in AHI are taken as the reference.*

	OSA variability category			
**Blood pressure variability**<br>OSA severity category	Quartile 1	Quartile 2	Quartile 3	Quartile 4
**Systolic**				
Mild OSA	0 (ref)	-0.04<br>(-0.32, 0.23)	0.18<br>(-0.10, 0.45)	0.51<br>(0.24, 0.80)
Moderate to severe OSA	0 (ref)	0.55<br>(0.22, 0.88)	0.59<br>(0.26, 0.93)	1.18<br>(0.85, 1.52)
**Diastolic**				
Mild OSA	0 (ref)	0.08<br>(-0.11, 0.27)	0.24<br>(0.05, 0.43)	0.40<br>(0.21, 0.60)
Moderate to severe OSA	0 (ref)	0.44<br>(0.22, 0.66)	0.44<br>(0.22, 0.66)	0.80<br>(0.57, 1.03)

Supplementary Table 13: *Estimated marginal means (mean and 95% confidence interval) of the association between obstructive sleep apnoea (OSA) severity and variability with diastolic and systolic blood pressure variability. Mild OSA is defined as an apnoea/hypopnoea index (AHI) between 5 and 15 events/h. Moderate/Severe OSA is defined as an AHI of more than 15 events/h. Within each OSA severity category, the lowest quartiles of variability in AHI are taken as the reference. Only blood pressure recordings taken in the morning were included in this analysis.*

	OSA variability			
**Blood pressure variability**<br>OSA severity category	**Quartile 1**	**Quartile 2**	**Quartile 3**	**Quartile 4**
**Systolic**				
Mild OSA	0 (ref)	0.15<br>(-0.25, 0.55)	-0.18<br>(-0.58, 0.22)	0.47<br>(0.06, 0.87)
Moderate to severe OSA	0 (ref)	0.23<br>(-0.22, 0.70)	0.50<br>(0.00, 0.90)	0.87<br>(0.41, 1.32)
**Diastolic**				
Mild OSA	0 (ref)	0.22<br>(-0.05, 0.50)	0.04<br>(-0.24, 0.31)	0.41<br>(0.13, 0.69)
Moderate to severe OSA	0 (ref)	0.13<br>(-0.18, 0.43)	0.34<br>(0.03, 0.65)	0.44<br>(0.13, 0.75)

Supplementary Figures

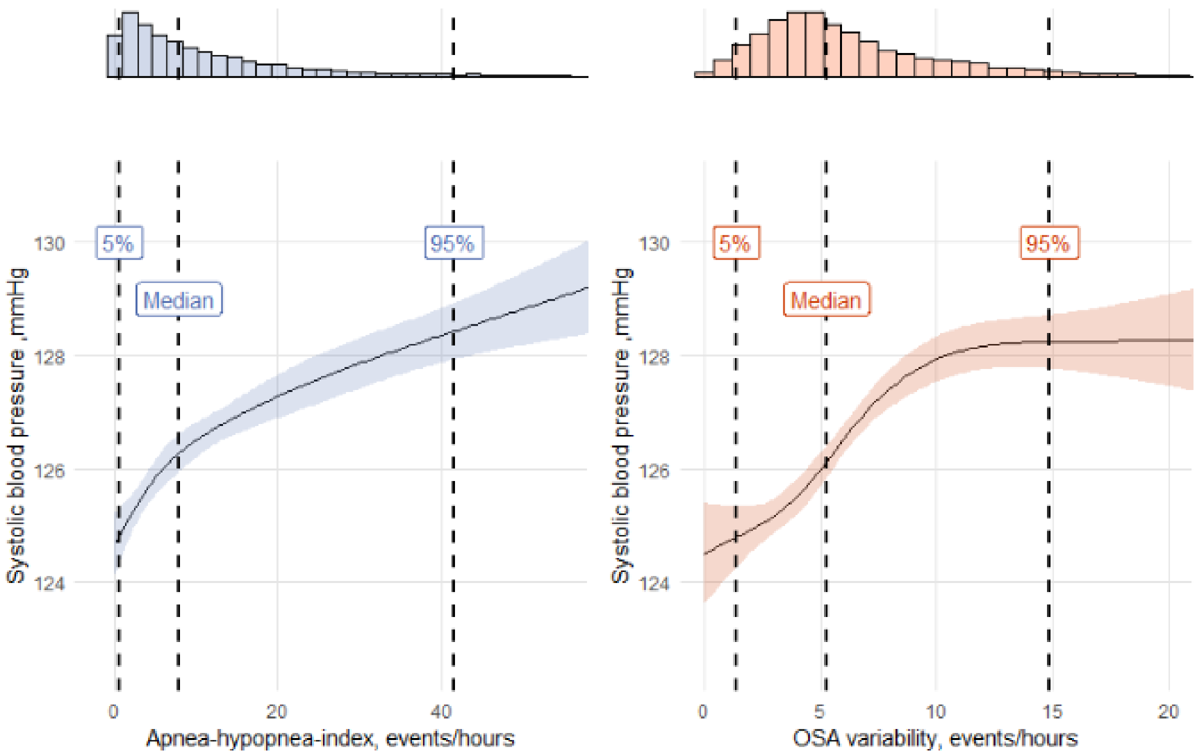

Supplementary Figure 1: Association between obstructive sleep apnoea (OSA) severity and variability with systolic blood pressure. *β coefficients (estimates and 95% confidence interval) of the association between obstructive sleep apnoea (OSA) severity and variability with systolic blood pressure, defined as the mean (left) and standard deviation of the apnoea-hypopnoea index (right), respectively. Models were adjusted for age, sex and BMI. Histograms represent the distribution of the average apnoea-hypopnoea-index (top left) and OSA variability (top right) for the entire population. Note: an increase in modelled systolic blood pressure with an increase in mean apnoea-hypopnoea index and with an increase in OSA variability.*

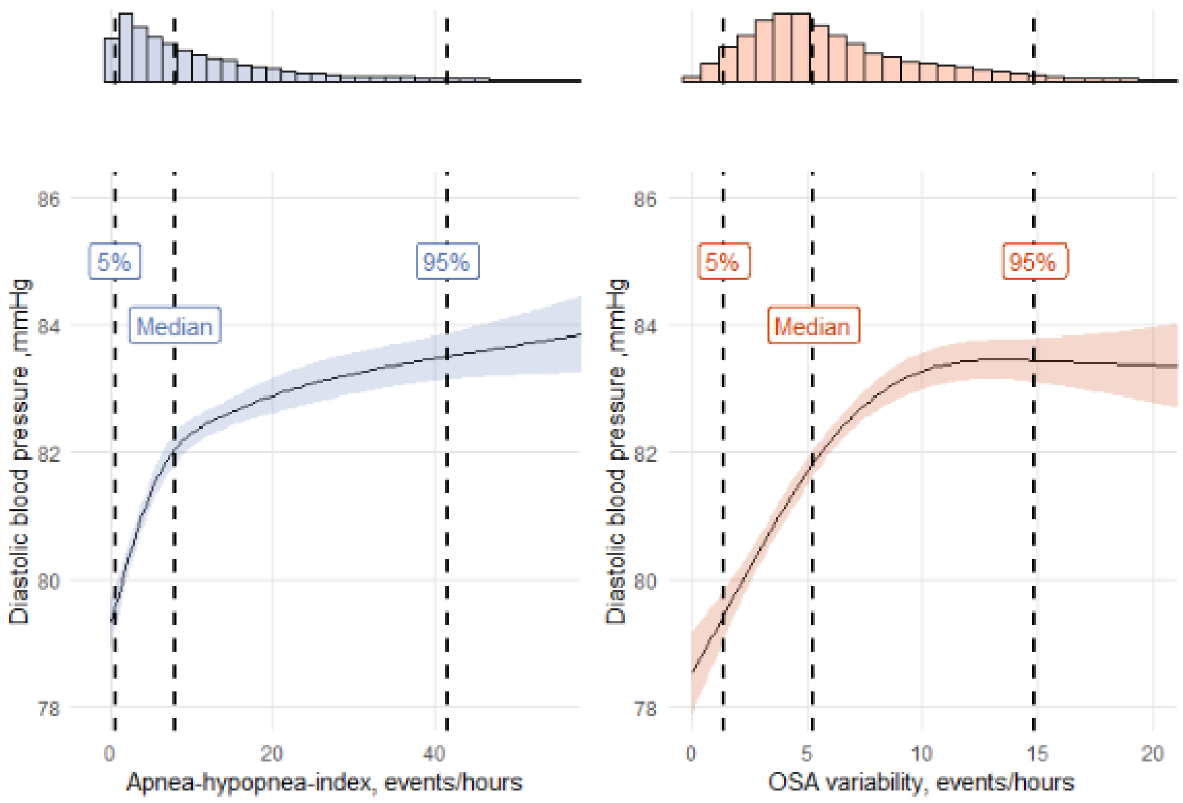

Supplementary Figure 2: Association between obstructive sleep apnoea (OSA) severity and variability with diastolic blood pressure. *β coefficients (estimates and 95% confidence interval) of the association between obstructive sleep apnoea (OSA) severity and variability with diastolic blood pressure, defined as the mean (left) and standard deviation of the apnoea-hypopnoea index (right), respectively. Models were adjusted for age, sex and body mass index. Histograms represent the distribution of the average apnoea-hypopnoea-index (top left) and OSA variability (top right) for the entire population. Note: an increase in modelled diastolic blood pressure with an increase in mean apnoea-hypopnoea index and with an increase in OSA variability.*

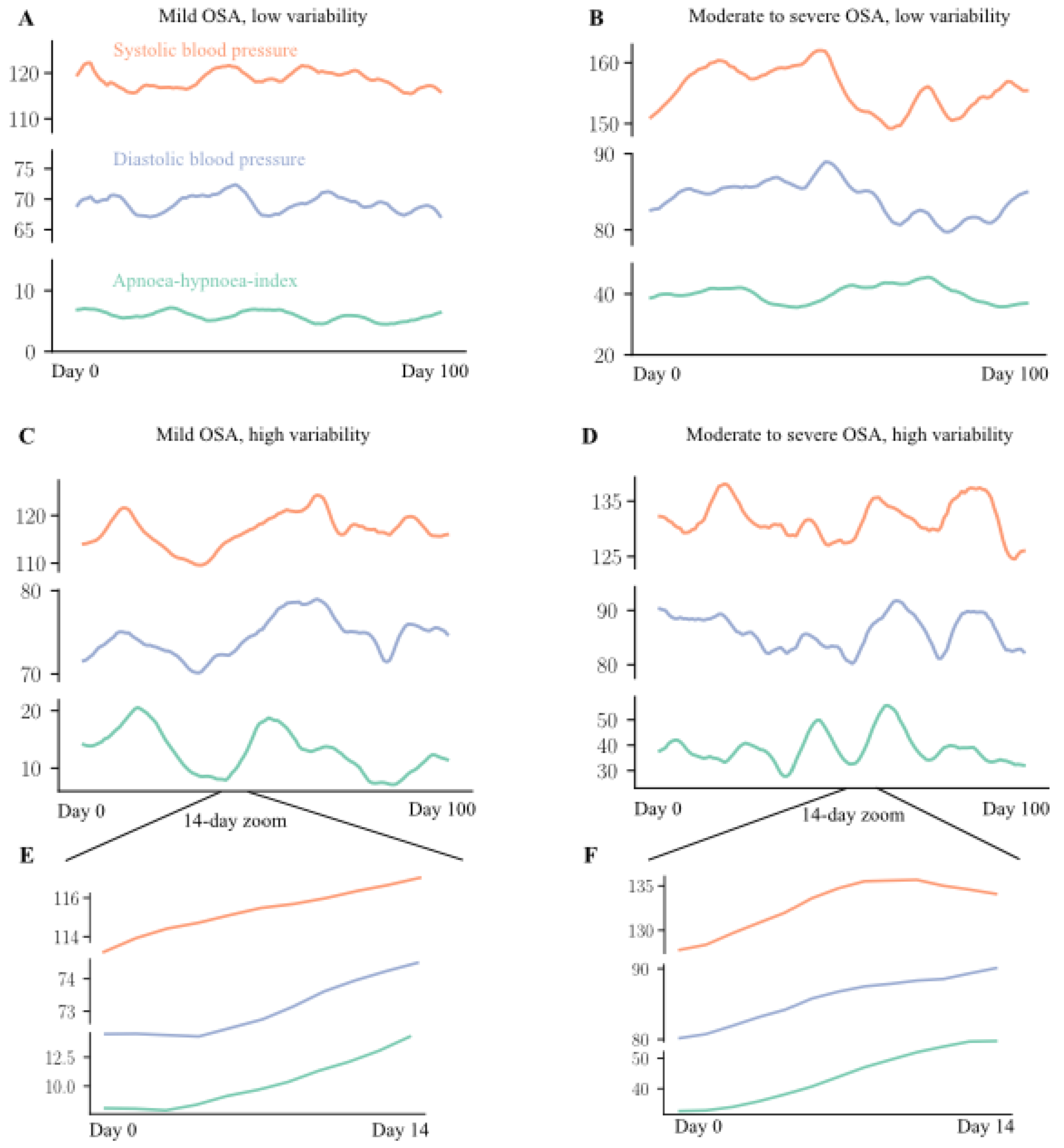

Supplementary Figure 3: Nightly variation systolic, diastolic blood pressure and estimated apnea-hypopnea index. *Nightly variations in diastolic (blue) and systolic blood pressure (orange) and apnoea-hypopnoea index (AHI; green) for a participant with mild obstructive sleep apnea (OSA; 5 < AHI < 15 events/h), low variability (A) or high variability (C). (B) Participant with moderate to severe OSA (AHI > 15 events/h) and low variability or high variability (D). (E) and (F) zoom on a 14-day period. Blood pressure measurements and apnoea-hypopnoea index were smoothed using a 14-day average rolling windows.*

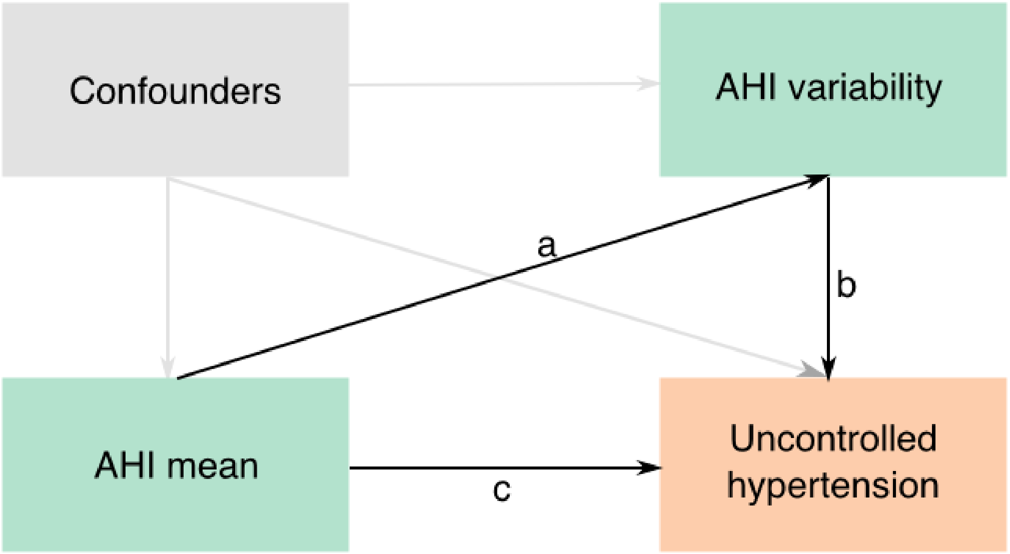

Supplementary Figure 4: *Mediation analysis. The direct effect of OSA severity (AHI mean) on uncontrolled hypertension is path c. The effect of OSA severity on OSA variability is path a, and the effect of OSA variability on uncontrolled hypertension is path b. Path a*b denotes the indirect effect of AHI mean on uncontrolled hypertension through a potential increase in night-to-night variability of OSA severity. The total effect of OSA severity on uncontrolled hypertension is c+ (a*b).*

Supplementary Discussion

There are important limitations associated with the mediation analysis performed here that need to be acknowledged. As highlighted in the main text, the effects of OSA severity and night-to-night variability of OSA severity on uncontrolled hypertension are non-linear. Whether there are true non-linear associations or whether the non-linear association is due to the mediation effect shown above is unknown. Furthermore, it is unknown whether this analysis represents a true mediation effect or whether OSA severity and night-to-night variability of OSA severity might both contribute to an overall construct of "severe OSA". Finally, there are potential confounding variables associated on the proposed pathways that are not accounted for (e.g., body and head position), which may limit the interpretations of these findings.

Supplementary References

1. Lechat B, Naik G, Reynolds A, Aishah A, Scott H, Loffler KA, Vakulin A, Escourrou P, McEvoy RD, Adams RJ, Catcheside PG, Eckert DJ. Multinight Prevalence, Variability, and Diagnostic Misclassification of Obstructive Sleep Apnea. *Am J Respir Crit Care Med* 2022: 205(5): 563-569.
2. Nieto FJ, Young TB, Lind BK, Shahar E, Samet JM, Redline S, D'Agostino RB, Newman AB, Lebowitz MD, Pickering TG. Association of sleep-disordered breathing, sleep apnea, and hypertension in a large community-based study. Sleep Heart Health Study. *JAMA* 2000: 283(14): 1829-1836.
3. Jones NR, McCormack T, Constanti M, McManus RJ. Diagnosis and management of hypertension in adults: NICE guideline update 2019. *Br J Gen Pract* 2020: 70(691): 90-91.
4. Stergiou GS, Palatini P, Parati G, O'Brien E, Januszewicz A, Lurbe E, Persu A, Mancia G, Kreutz R, European Society of Hypertension C, the European Society of Hypertension Working Group on Blood Pressure M, Cardiovascular V. 2021 European Society of Hypertension practice guidelines for office and out-of-office blood pressure measurement. *J Hypertens* 2021: 39(7): 1293-1302.
5. Whelton PK, Carey RM, Aronow WS, Casey DE, Jr., Collins KJ, Dennison Himmelfarb C, DePalma SM, Gidding S, Jamerson KA, Jones DW, MacLaughlin EJ, Muntner P, Ovbiagele B, Smith SC, Jr., Spencer CC, Stafford RS, Taler SJ, Thomas RJ, Williams KA, Sr., Williamson JD, Wright JT, Jr. 2017 ACC/AHA/AAPA/ABC/ACPM/AGS/APhA/ASH/ASPC/NMA/PCNA Guideline for the Prevention, Detection, Evaluation, and Management of High Blood Pressure in Adults: A Report of the American College of Cardiology/American Heart Association Task Force on Clinical Practice Guidelines. *J Am Coll Cardiol* 2018: 71(19): e127-e248.

6. Muntner P, Shimbo D, Carey RM, Charleston JB, Gaillard T, Misra S, Myers MG, Ogedegbe G, Schwartz JE, Townsend RR, Urbina EM, Viera AJ, White WB, Wright JT, Jr. Measurement of Blood Pressure in Humans: A Scientific Statement From the American Heart Association. *Hypertension* 2019: 73(5): e35-e66.
