# Supplementary material for: High night-to-night variability in sleep apnea severity is associated with uncontrolled hypertension
Source: NPJ Digit Med. 2023 Mar 30;6:57. doi: 10.1038/s41746-023-00801-2 (PMC10060245; doi:10.1038/s41746-023-00801-2)
Supplement: Supplementary file 2 — REPORTING SUMMARY [file 41746_2023_801_MOESM2_ESM.pdf]

nature portfolio

Corresponding author(s): Bastien Lechat

Last updated by author(s): 25/01/2023

Reporting Summary

Nature Portfolio wishes to improve the reproducibility of the work that we publish. This form provides structure for consistency and transparency in reporting. For further information on Nature Portfolio policies, see our Editorial Policies and the Editorial Policy Checklist.

Statistics

For all statistical analyses, confirm that the following items are present in the figure legend, table legend, main text, or Methods section.

n/a	Confirmed	
☐	☒	The exact sample size (*n*) for each experimental group/condition, given as a discrete number and unit of measurement
☐	☒	A statement on whether measurements were taken from distinct samples or whether the same sample was measured repeatedly
☐	☒	The statistical test(s) used AND whether they are one- or two-sided<br>*Only common tests should be described solely by name; describe more complex techniques in the Methods section.*
☐	☒	A description of all covariates tested
☐	☒	A description of any assumptions or corrections, such as tests of normality and adjustment for multiple comparisons
☐	☒	A full description of the statistical parameters including central tendency (e.g. means) or other basic estimates (e.g. regression coefficient) AND variation (e.g. standard deviation) or associated estimates of uncertainty (e.g. confidence intervals)
☒	☐	For null hypothesis testing, the test statistic (e.g. *F*, *t*, *r*) with confidence intervals, effect sizes, degrees of freedom and *P* value noted<br>*Give P values as exact values whenever suitable.*
☒	☐	For Bayesian analysis, information on the choice of priors and Markov chain Monte Carlo settings
☒	☐	For hierarchical and complex designs, identification of the appropriate level for tests and full reporting of outcomes
☒	☐	Estimates of effect sizes (e.g. Cohen's *d*, Pearson's *r*), indicating how they were calculated

Our web collection on statistics for biologists contains articles on many of the points above.

Software and code

Policy information about availability of computer code

Data collection	not applicable
Data analysis	Logistic and linear regression was performed in the R programming language, using the rms modeling package [28].<br><br>[28] Harrell EF. Regression Modeling Strategies. Springer International Publishing, Cham, Switzerland, 2015.

For manuscripts utilizing custom algorithms or software that are central to the research but not yet described in published literature, software must be made available to editors and reviewers. We strongly encourage code deposition in a community repository (e.g. GitHub). See the Nature Portfolio guidelines for submitting code & software for further information.

Data

Policy information about availability of data

All manuscripts must include a data availability statement. This statement should provide the following information, where applicable:

- Accession codes, unique identifiers, or web links for publicly available datasets
- A description of any restrictions on data availability
- For clinical datasets or third party data, please ensure that the statement adheres to our policy

Deidentified data that support the findings of this study, including individual data, are available from the corresponding author upon request subject to ethical and data custodian (Withings) approval.

Human research participants

Policy information about studies involving human research participants and Sex and Gender in Research.

Reporting on sex and gender	We have use sex throughout the manuscript. No information on gender was available.
Population characteristics	All users in this study were prompted to enter their age, sex, height, and weight from which body mass index (BMI) is calculated.
Recruitment	Data were acquired retrospectively from 15,526 participants from a consumer-user database of people aged between 18-90 years who purchased an under-mattress sleep sensor device (Withings Sleep Analyzer: WSA) and reported blood pressure measurements on at least 5 separate occasions.
Ethics oversight	The current study was approved by the Flinders University Human Research Ethics Committee

Note that full information on the approval of the study protocol must also be provided in the manuscript.

Field-specific reporting

Please select the one below that is the best fit for your research. If you are not sure, read the appropriate sections before making your selection.

☒ Life sciences ☐ Behavioural & social sciences ☐ Ecological, evolutionary & environmental sciences

For a reference copy of the document with all sections, see nature.com/documents/nr-reporting-summary-flat.pdf

Life sciences study design

All studies must disclose on these points even when the disclosure is negative.

Sample size	This is a retrospective analysis of a consumer-database.
Data exclusions	Participant were excluded if they did not use the under-mattress-sensors (WSA) enough. Specifically, inclusion criteria included ≥28 nights of WSA recordings and an average use of ≥4 times per week. Participant were also excluded if they had less than 5 blood pressure recordings (see methods section of the manuscript under "participant" heading)
Replication	This is an observational study of a retrospective database hence there was no experimental findings.
Randomization	This is an observational study, thus there was no randomization.
Blinding	This is a retrospective analysis of a consumer-user database, thus the investigator did not participate in data collection

Reporting for specific materials, systems and methods

We require information from authors about some types of materials, experimental systems and methods used in many studies. Here, indicate whether each material, system or method listed is relevant to your study. If you are not sure if a list item applies to your research, read the appropriate section before selecting a response.

Materials & experimental systems

n/a	Involved in the study	
☒	☐	Antibodies
☒	☐	Eukaryotic cell lines
☒	☐	Palaeontology and archaeology
☒	☐	Animals and other organisms
☒	☐	Clinical data
☒	☐	Dual use research of concern

Methods

n/a	Involved in the study	
☒	☐	ChIP-seq
☒	☐	Flow cytometry
☒	☐	MRI-based neuroimaging
